# Supplementary material for: BoHV-1-Vectored BVDV-2 Subunit Vaccine Induces BVDV Cross-Reactive Cellular Immune Responses and Protects against BVDV-2 Challenge
Source: Vaccines (Basel). 2021 Jan 13;9(1):46. doi: 10.3390/vaccines9010046 (PMC7828602; doi:10.3390/vaccines9010046)
Supplement: Supplementary file 1 [file vaccines-09-00046-s001.pdf]

BVDV-2 E2

5′ – KpnI hEF1-α promoter

GGTACCCTCGTGAGGCTCCGGTGCCCGTCAGTGGGCAGAGCGCACATCGCCCACAGTCCCCGAGAAGTTG
GGGGGAGGGGTCGGCAATTGAACCGGTGCCTAGAGAAGGTGGCGCGGGGTAAACTGGGAAAGTGATGTCG
TGTACTGGCTCCGCCTTTTTCCCGAGGGTGGGGGAGAACCGTATATAAGTGCAGTAGTCGCCGTGAACGT
TCTTTTTCGCAACGGGTTTGCCGCCAGAACACAGGTAAGTGCCGTGTGTGGTTCCCGCGGGCCTGGCCTC
TTTACGGGTTATGGCCCTTGCGTGCCTTGAATTACTTCCACCTGGCTGCAGTACGTGATTCTTGATCCCG
AGCTTCGGGTTGGAAGTGGGTGGGAGAGTTCGAGGCCTTGCGCTTAAGGAGCCCCTTCGCCTCGTGCTTG
AGTTGAGGCCTGGCCTGGGCGCTGGGGCCGCCGCGTGCGAATCTGGTGGCACCTTCGCGCCTGTCTCGCT
GCTTTCGATAAGTCTCTAGCCATTTAAAATTTTTGATGACCTGCTGCGACGCTTTTTTTCTGGCAAGATA
GTCTTGTAAATGCGGGCCAAGATCTGCACACTGGTATTTCGGTTTTTGGGGCCGCGGGCGGCGACGGGGC
CCGTGCGTCCCAGCGCACATGTTCGGCGAGGCGGGGCCTGCGAGCGCGGCCACCGAGAATCGGACGGGGG
TAGTCTCAAGCTGGCCGGCCTGCTCTGGTGCCTGGCCTCGCGCCGCCGTGTATCGCCCCGCCCTGGGCGG
CAAGGCTGGCCCGGTCGGCACCAGTTGCGTGAGCGGAAAGATGGCCGCTTCCCGGCCCTGCTGCAGGGAG
CTCAAAATGGAGGACGCGGCGCTCGGGAGAGCGGGCGGGTGAGTCACCCACACAAAGGAAAAGGGCCTTT
CCGTCCTCAGCCGTCGCTTCATGTGACTCCACGGAGTACCGGGCGCCGTCCAGGCACCTCGATTAGTTCT
CGAGCTTTTGGAGTACGTCGTCTTTAGGTTGGGGGGAGGGGTTTTATGCGATGGAGTTTCCCCACACTGA
GTGGGTGGAGACTGAAGTTAGGCCAGCTTGGCACTTGATGTAATTCTCCTTGGAATTTGCCCTTTTTGAG
TTTGGATCTTGGTTCATTCTCAAGCCTCAGACAGTGGTTCAAAGTTTTTTTCTTCCATTTCAGGTGTCGT
GAGGAATTAGCTTGGTACTAATACGACTCACTATAGGGAGACCCAAGCTGGCTAGGTAAGTGTACGAGCT
CGATCACTAGTCCAGTGTGG**atcgat***CGCCGCCACC*ATGCAGGGACCAACACTGGCCGTGCTGGGGGCTC

ClaI Kozak BHV-1 gD signal sequence

TGCTGGCTGTGGCTGTCTCCCTGTTCCCCGAGTGCAAGGAAGGATTTCAGTACGCCATCAGCAAGGACCG
GAAAATTGGACCACTGGGACCAGAGTCCCTGACCACAACTTGGCACCTGCCCACCAAGAAAATCGTGGAC
TCTATGGTGCAGGTCTGGTGCGATGGCAAGAACCTGAAAATTCTGGAGACATGTACTAAGGAGGAGAGAT
ACCTGGTGGCTGTCCACGAGCGCGCTCTGTCTACCAGTGCCGAGTTCATGCAGATCAGCTCCGGAACAAA
GGGCCCTGAAGTGATCGACATGCACGACGATTTCGAATTTGGCCTGTGCCCCTGTGATAGTAAGCCTGTG
ATGCGCGGAAAATTCAACGCTTCACTGCTGAATGGCCCTGCCTTTCAGATGGTGTGCCCACAGGGGTGGA
CCGGAACAATCGAGTGTATTCTGGCTAACCAGGACACACTGGATACCACAGTGGTCCGGACTTACCGGAG
GACTACCCCTTTTCAGCGCAGAAAGTGGTGCACCTATGAGAAAATCATTGGCGAGGACATCCACGAGTGC
ATCCTGGGCGGGAATTGGACCTGTATCACAGGCGACCATTCTAAGCTGAAAGATGGGCCAATTAAGAAAT
GCAAGTGGTGTGGCTACGACTTCTTTGATAGTGAGGGACTGCCTCATTATCCAATCGGCAAATGTATGCT
GTCAAACGAAAGCGGGTACAGATATGTGGACGATACTAGCTGCGATCGAGGAGGAGTGGCTATCGTCCCA
ACTGGGACCCTGAAGTGTAGGATCGGAAAAGCTACCGTGCAGGTCATTGCCACAAATACTGACCTGGGAC
CAATGCCTTGCTCCCCAGATGAAGTGATCGCTTCTGAGGGACCTGTCGAAAAGACTGCCTGTACCTTCAA
CTACTCCAAGACACTGCCAAACAAGTACTATGAGCCCCGAGACCGGTACTTCCAGCAGTATATGCTGAAG
GGGGAATGGCAGTACTGGTTTGACCTGGATACCGTGGACCACCATAAGGATTACTTCTCAGAGTTTATCG
TGATTGCCGTGGTCGCTCTGCTGGGGGGAAAGTACGTGCTGTGGCTGCTGGTCACCTATATGATCCTGAG
TGAACAGATGGCCATGGGC**atgcat***ggtaagcctatccctaaccctctcctcggtctcgattctacgcgt*

NsiI V5 epitope

*accggt***CATCATCACCATCACCAT*****TGA****GTTTAAACCCGCTGATCAGCCTCGACTGTGCCTTCTAGTTGCC*

6xHis ***Stop*** *BGH Poly A*

AGCCATCTGTTGTTTGCCCCTCCCCCGTGCCTTCCTTGACCCTGGAAGGTGCCACTCCCACTGTCCTTTC
CTAATAAAATGAGGAAATTGCATCGCATTGTCTGAGTAGGTGTCATTCTATTCTGGGGGGTGGGGTGGGG
*CAGGACAGCAAGGGGGAGGATTGGGAAGACAATAGCAGGCATGCTGGGGATGCGGTGGGCTCTATGG*CTT
GGTACC–3'

KpnI

Supplementary Fig. 1. Nucleotide sequence of chimeric BVDV-2 E2 gene cassette. BVDV-2 E2 chimeric ORF sequence contained within ClaI and NsiI sites were designed to include 5'-3' the following: Kozak sequence, codon optimized for Bos Taurus (Genscript) nucleotide sequence of predicted BHV-1 gD signal sequence (GenBank accession # AFV53430.1, aa 1-19), BVDV E2-2 coding

nucleotide (GenBank accession # AAC72814.1). The 1.195 bp ClaI/NsiI fragment was cloned into the corresponding ClaI/NsiI sites of the pPreBVDV-E2 chimeric vector (sequence file 1) resulting in BVDV-2 E2 chimeric gene expression cassette. BVDV-2 E2 sequence is indicated by uppercase underlined and with green letters.

5’- NotI

GCGGCCGCaggcaacggggcctgctcccgcgcaggagcacgtggtgctcaagatcggggcctcggcctct

BHV-1 gG flanking upstream

acgctggccgaggctatgctactgcgaaccttggaccacgccaacgtggtcaagctgaaggccgtgctct
tccacggggagctggtgtgcgtggtgctggcgcgctaccgcgaggacctgcacacgcacctctggagaat
caaccgcccgctggcgctccccgcggcgctggcggtgacgcgggccgtgctgcggggcctcgcgtacctg
cactcccgccggatcgctcaccgggacgtcaaaacggaaaacgtcttcctcaacggcccaggcgacgtgt
gcctgggcgactttggcgcggcacacgggccggtcaccgagccccgctactacggcctggccggcaccct
ggagacgaactcgccagagctgctggcgcgcgcgcgctacgactgccgcacggacgtgtggagcgcgggc
gtcgtcgcgtacgagatgctggcatacccgcgcgcgctgttcgacagccccgcgggcccgcagggcgagg
acgccgaggcatcgggcccgccgacgatcttgggcgaccgcgactgcgcccggcagctgctccgcgtgat
tcgccggctggccgtgcacgccgaagagtttccacccagccccactgaccggctgacccgcaacttcaag
cgccacgcgagcacgcgccgagagccgcacagcccgtaccgctgcctggcggtgctccggctgccctgcg
acgccgaccgcctcctacaccagatgctgacctttgactttcgcgcgcgccccaccgccgcggagctgct
ggagcaccccgtcttcggtgcggcctcggggtagccccgggggtttcccgcaaaactgaggcatataagg
cgcgggcaccggcaagtttggcatccacacttcgcgctgtggacacgagagcgaacgcgagcgaacgcga
gcgcaagcgcgagcacacgactgcgatc***tacaataaacag*****GGTACC***TAGTTATTAATAGTAATCAATTAC*

Chimeric Us3/Poly A KpnI *CMV promotor*

GGGGTCATTAGTTCATAGCCCATATATGGAGTTCCGCGTTACATAACTTACGGTAAATGGCCCGCCTGGC
TGACCGCCCAACGACCCCCGCCCATTGACGTCAATAATGACGTATGTTCCCATAGTAACGCCAATAGGGA
CTTTCCATTGACGTCAATGGGTGGAGTATTTACGGTAAACTGCCCACTTGGCAGTACATCAAGTGTATCA
TATGCCAAGTACGCCCCCTATTGACGTCAATGACGGTAAATGGCCCGCCTGGCATTATGCCCAGTACATG
ACCTTATGGGACTTTCCTACTTGGCAGTACATCTACGTATTAGTCATCGCTATTACCATGGTGATGCGGT
TTTGGCAGTACATCAATGGGCGTGGATAGCGGTTTGACTCACGGGGATTTCCAAGTCTCCACCCCATTGA
CGTCAATGGGAGTTTGTTTTGGCACCAAAATCAACGGGACTTTCCAAAATGTCGTAACAACTCCGCCCCA
TTGACGCAAATGGGCGGTAGGCGTGTACGGTGGGAGGTCTATATAAGCAGAGCTGGTTTAGTGAACCGTC
*AGATCCGCTAGCGCTACCGGT***GCCGCCACC**ATGCAGGGACCTACTCTGGCTGTGCTGGGGGCTCTGCTGG

Kozak BHV-1 gD signal

CTGTGGCTGTCTCTCTGGAAAACATTACTCAGTGGAATCTGATGGACAATGGCACCGAAGGGATCCAGCA

BVDV 2 E^{rns}

GGCCATGTTTCTGCGAGGCGTGAACCGGTCTCTGCACGGGATCTGGCCTGAGAAGATTTGCACCGGCGTG
CCAACACATCTGGCCACTGACTACGAGCTGAAGGAAATTGTCGGGATGATGGATGCTAGTGAGAAAACTA
ATTATACCTGCTGTCGCCTGCAGAGACACGAATGGAACAAGCATGGGTGGTGTAATTGGTTCCACATCGA
GCCATGGATTTGGCTGATGAACAAAACACAGAACAATCTGACTGAGGGACAGCCACTGAGGGAATGCGCT
GTGACCTGTCGCTACGACAAGGAGACCGAACTGAATATCGTCACACAGGCTCGGGATAGGCCTACCACAC
TGACCGGCTGCAAGAAAGGGAAAAACTTCAGCTTTGCCGGCGTGATCCTGGACGGACCATGCAACTTCAA
GGTGAGCGTCGAGGACGTGCTGTTCAAGGAACACGATTGTGGAAACATGCTGCAGGAAACAGCCATCCAG
CTGCTGGATGGCGCTACAAATACTATTGAGGGAGCTCGAGTGGGAACCGCTAAGCTGACTACCTGGCTGG
GCAAACAGCTGGGAATCCTGGGCAAGAAACTGGAGAACAAGTCTAAAGCCTGGTTTGGGGCCCATGCTAT

GM-CSF

GGCCCCCACTAGACCCCCTAACACCGCTACACGACCTTGGCAGCACGTGGACGCCATTAAGGAGGCTCTG
AGCCTGCTGAACCATAGCTCCGACACTGATGCCGTGATGAATGACACCGAGGTGGTCTCCGAAAAGTTTG
ATTCTCAGGAGCCCACCTGTCTGCAGACACGGCTGAAGCTGTACAAAAACGGGCTGCAGGGATCACTGAC
CAGCCTGATGGGATCCCTGACTATGATGGCTACCCACTATGAGAAGCATTGCCCACCCACACCTGAAACT

AGTTGTGGCACCCAGTTCATCAGCTTCAAGAACTTCAAAGAAGACCTGAAAGAGTTCCTGTTTATTATTC
CATTTGACTGTTGGGAGCCTGCCCAGAAG*gactacaaagacgatgacgacaag****TAA***CTGATCATAATCAG*

Flag epitope **Stop** SV40 terminator

CCATACCACATTTGTAGAGGTTTTACTTGCTTTAAAAAACCTCCCACACCTCCCCCTGAACCTGAAACAT
AAAATGAATGCAATTGTTGTTGTTAACTTGTTTATTGCAGCTTATAATGGTTACAAATAAAGCAATAGCA
TCACAAATTTCACAAATAAAGCATTTTTTTCACTGCATTCTAGTTGTGGTTTGTCCAAACTCATCAATGT
*ATCTTA***AAGCTT**ggacgcttcgcgcggctgcgccctggccctcttggacatggcggagacggtggtgccc

HindIII BHV-1 gG flanking downstream

ggcggaccgcgagccgccgacgtcgtcgacgtcggctgggcttaccaagacggggactgcatggtgcctc
tggcatatcgccagtactttaactgcacgggggcgcgctgcccggccaaaacgtctgcgccgggctctc
tgagacccgcatccgcggtggctttggaacctccgactacgcgctctacgggacgtcgctagtactgcgc
cccggcctgtacgaccgcgggacctacatctacttccttggatacggcccagacgacatctacgtgggca
gcgtcacgctcatggtgggcgccgacatccacaaataccccctgcgggctggaccgagggctcggtgtggc
cctgcaccacaagagcggaccggcccgacctctgacagaggacgacgccaccggcgactgggcctgcggc
tgcttccccgcccttgttgaggttgacgcggtgtggggcaacgtaagcgccgcagagctgggcctggccg
acccgatcgactacgccgacgaagggggtgaggtcgaagtgctcgaggacgaagccgggagcgccagcgg
aaacctgccgcaggacgaccccgaccccgacctcgcagattgccggaccgtcgggctctttagcgaaagc
gacatgttccggaccgccagcgggcccgaatcgctgctgatcggcgccgttgccaaggacgtcctgacgg
tgcccctcaatctgccgcccggccgctcttacgaggccctgcgaaacgcatcgctggagtgcaactcccg
cccgcgcgagaccggcgacgcagcggtggtggtgatgtctctccaggagcccgctcgcctcgagcgccgc
cccgatgcccgcgccaccgatccggagtttgggctctttggcctgcccgatgaccccgccgtgcggcgcg
gcattctcatcggcctcgcgatcgctctgctg**ATGCAT**

NsiI – 3’

Supplementary Fig. 2: Nucleotide sequence of chimeric BVDV-2 E^{rns} GM-CSF-Flag gene insertion plasmid (pBVD2-E2.INS**).** The 4028 bp long nucleotide sequence for the BHV-1 gGΔ BVDV2 E^{rns} GM-CSF Flag insertion was assembled using the plasmid pBHV-1 gGΔ and pBVDV2 E^{rns} GM-CSF chimeric gene cassette (p). The BHV-1 gGΔ BVDV-2 E^{rns} GM-CSF insertion plasmid consisted of the following (5’ to 3’): i) NotI restriction site (bold, underlined); ii) BHV-1 gG flanking upstream (lower case); iii) Us3/Us4 polyadenylation signal (lower case, bold, italic); iv) KpnI restriction site (bold, underlined); v) CMV promotor sequence (italic) vi) Kozak sequence (bold); vii) BHV-1 gD signal (highlighted); viii) BVDV-2 E^{rns} coding sequence; ix) GM-CSF coding sequence (underlined, highlighted); x) Flag epitope coding sequence (lower case, italic); xi) Stop codon (TAA) (bold, italic); xii) SV40 terminator (italic, underlined); xiii) HindIII restriction site (bold, underlined); xiv) BHV-1 gG flanking downstream (lower case); NsiI restriction site (bold, underlined).

Supplementary Table 1. **Clinical scoring scheme**

Clinical Scoring criteria	Rectal Temp [°C]	Nasal/Ocular discharge	Lethargy	Diarrhea	Dyspnea	Mucosal or oral lesions	Anorexia	Cough
Normal (0)	< 39.5	None	Normal in attitude and activities	Normal feces	Normal breathing	No nasal or oral lesions	Normal appetite	None
Mild (1)	39.5-39.9	Serous discharge	Moves slowly, head down	Soft, slightly loose feces	Slight difficulty breathing, short and rapid	White colored lesions	Slightly off feed	<3 episodes occasional cough
Moderate (2)	40-40.4	Mucopurulent discharge	Tends to lie down, moves reluctantly	Watery diarrhea	Labored breathing, noticeable abdominal	Red lesions	Moderately off feed	>3 episodes repeated cough
Severe (3)	40.5-40.9	Copious mucopurulent discharge	Stands with difficulty or not at all, little or no interest in surroundings or response to stimuli	Watery and bloody diarrhea	Very labored, grunting or raspy breathing	Bloody, ulcerated lesions	Not eating	NA
(4)	>40.9	NA	NA	NA	NA	NA	NA	NA

Group	Animal #	Serum neutralizing antibody response against BVDV2 125<br>Days post vaccination/ Days post challenge																	
		0	Mean	7	Mean	14	Mean	21	Mean	28	Mean	34/0	Mean	40/6	Mean	48/14	Mean	54/20	Mean
	626	4		4		4		4		4		4		4		512		1,024	
	636	16		8		4		4		4		4		4		1,024		16,384	
Control	637	4	31	4	30	4	16	4	16	4	10	4	10	4	6	512	1,638	2,048	5,939
	638	4		4		4		4		4		4		4		2,048		2,048	
	647	128		128		64		64		32		32		16		4,096		8,192	
	627	4		4		4		4		4		8		8		1,024		4,096	
	628	4		4		4		4		64		256		256		32,768		32,768	
Bovi	629	4	4	4	4	4	4	4	118	4	91	32	878	64	885	32,768	16,589	16,384	15,565
	632	4		4		4		512		256		2,048		2,048		8,192		8,192	
	635	4		4		4		64		128		2,048		2,048		8,192		16,384	
	630	4		4		4		4		4		4		64		262,144		65,536	
QMV – BVD2*	633	4	4	4	4	4	4	4	4	4	5	16	17	64	64	65,536	94,208	65,536	45,056
	639	4		4		4		4		8		16		64		32,768		16,384	
	640	4		4		4		4		4		32		64		16,384		32,768	

Supplementary Table 2: **BVDV-2-specific serum neutralizing antibody response.**

Group	Animal #	Leukocyte count [10³/uL]													
		Days post-vaccination/ Days post-challenge													
		0 dpv	Mean	34/0	Mean	38/4	Mean	40/6	Mean	42/8	Mean	45/11	Mean	48/14	Mean
Control	626	14.00	15.5	10.10	11.58	7.00	8.1	5.50	6.2	8.4	7.74	7.0	7.26	7.2	8.6
	636	21.10		10.70		8.50		6.4		5.20		6.1		5.4	
	637	13.50		13.40		8.60		6.60		7.2		6.5		8.9	
	638	19.10		8.50		7.50		6.00		9.9		7.2		9.2	
	647	9.80		15.20		8.90		6.70		8.0		9.5		12.3	
Bovi	627	18.60	15.94	13.30	13.02	10.00	11.9	13.7	13.54	14.6	13.04	16.7	14.02	16.9	13.18
	628	15.70		11.10		10.70		14.2		11.3		11.2		8.8	
	629	17.60		10.60		10.50		14.3		14.5		14.6		13.6	
	632	13.20		15.60		14.70		13.9		13.10		13.2		13.8	
	635	14.60		14.50		13.60		11.60		11.7		14.4		12.8	
QMV-BVD2*	630	14.60	13.35	6.90	10.37	4.80	7.42	4.20	8.92	10.9	10.78	8.2	9.82	8.1	9.4
	633	11.70		9.20		6.50		7.90		7.8		8.8		8.5	
	639	14.70		11.70		8.50		10.7		11.8		11.0		10.5	
	640	12.40		13.70		9.90		12.9		12.6		11.3		10.5	

Supplementary Table 3: **Leukocyte counts in animals for each treatment group at indicated days post –vaccination and post-challenge.**

Group	Animal #	Nasal shedding of BVDV-2 challenge virus - Days post-challenge (virus titer; PFUs/ml).									
		0	Mean	4	Mean	6	Mean	8	Mean	11	Mean
Control	626	0	0	5	48	90	140	25	66	0	1
	636	0		40		45		115		5	
	637	0		0		5		45		0	
	638	0		185		555		125		0	
	647	0		10		5		20		0	
Bovi	627	0	0	0	0	0	0	0	0	0	0
	628	0		0		0		0		0	
	629	0		0		0		0		0	
	632	0		0		0		0		0	
	635	0		0		0		0		0	
QMV – BVD2*	630	0	0	5	1.25	35	8.75	0	0	0	0
	633	0		0		0		0		0	
	639	0		0		0		0		0	
	640	0		0		0		0		0	

Supplementary Table 4: BVDV-2 nasal virus shedding following challenge

Group	Animal #	BVDV copies/ng of total RNA (log10) - Days post challenge									
		0	Mean	4	Mean	6	Mean	8	Mean	11	Mean
Control	626	1.443	1.362	5.109	13.302	150.985	372.260	18.140	677.602	4.298	47.184
	636	1.064		15.965		865.052		2660.792		136.072	
	637	1.570		10.307		186.098		551.371		78.710	
	638	1.370		18.677		486.931		108.324		10.519	
	647	1.367		16.454		172.237		49.386		6.324	
Bovi	627	0.703	1.158	2.098	1.712	2.334	2.294	1.806	2.276	1.713	1.739
	628	1.958		1.793		2.645		2.597		1.790	
	629	0		1.156		0.672		1.617		1.154	
	632	1.698		1.826		3.000		3.602		2.134	
	635	1.434		1.690		2.820		1.762		1.907	
QMV – BVD2*	630	1.0337	1.023	13.926	5.881	222.848	57.971	20.044	6.523	2.171	1.726
	633	0.819		2.534		2.288		1.753		1.520	
	639	1.104		2.982		2.103		1.613		1.675	
	640	1.138		4.082		4.645		2.685		1.541	

Table 5: **BVDV-2 viremia after challenge**.

Group	Animal number	Rectal temperature (°C)																					
		Days post vaccination / Days post challenge																					
		0	2	4	6	9	14	21	28	34/0	36/2	37/3	38/4	39/5	40/6	41/7	42/8	43/9	44/10	45/11	46/12	47/13	48/14
Control	#626	38.9	39.6	38.4	38.8	39.3	39.2	38.8	38.7	39.4	39.3	39.8	39.3	39.6	39.2	39.5	40.5	39.8	39.7	39.6	39.2	39.0	38.8
	#636	39.7	39.2	39.3	39.6	39.2	39.6	38.8	39.3	40.1	39.9	40.0	40.1	39.6	40.0	39.7	40.7	40.7	41.3	39.9	39.7	39.2	39.0
	#637	39.1	38.4	38.7	38.5	39.4	39.1	38.6	39.2	39.5	39.1	39.5	39.4	39.1	39.7	40.0	40.9	39.7	40.2	39.0	38.7	38.7	38.8
	#638	39.4	39.1	38.6	38.6	38.7	38.6	38.1	38.1	40.3	39.8	40.0	39.9	39.7	39.4	40.5	40.8	39.8	39.7	38.8	38.6	38.9	38.7
	#647	39.4	39.0	38.8	39.1	38.8	39.2	39.2	38.4	39.0	39.8	39.7	39.7	39.2	39.8	39.7	40.1	39.7	39.9	39.3	38.9	38.9	38.9
Bovi	#627	39.8	39.3	39.7	39.3	39.2	38.7	38.9	38.5	39.2	39.1	39.4	39.3	39.2	39.1	40.3	39.1	39.2	39.6	39.2	38.4	38.7	38.9
	#628	40.2	39.6	40.0	39.7	40.2	39.3	38.8	39.2	39.8	39.6	40.0	40.1	39.7	39.6	39.0	39.1	39.4	39.6	39.4	38.9	39.1	38.9
	#629	38.6	39.1	39.4	39.3	39.4	38.9	38.9	39.2	39.8	39.8	39.7	39.8	39.2	39.2	38.8	39.0	38.9	39.4	38.8	38.8	38.7	38.9
	#632	40.5	39.0	39.4	39.6	39.1	39.2	36.7	39.0	39.6	39.7	39.1	39.2	39.4	39.0	38.8	38.8	39.1	39.3	39.2	38.7	38.8	38.6
	#635	39.9	39.7	39.1	39.2	39.6	39.3	39.1	38.9	38.5	39.4	39.2	39.2	39.2	39.2	38.9	38.8	39.2	39.1	38.9	38.9	39.2	38.9
QMV-BVD2*	#630	38.9	39.0	39.1	37.6	38.5	38.8	38.8	39.2	39.1	38.9	39.3	39.6	39.8	39.6	38.9	40.0	38.9	39.1	38.7	38.6	38.6	38.6
	#633	39.4	39.4	38.8	38.9	38.9	39.3	38.6	38.6	39.6	39.4	39.3	39.3	39.2	39.4	39.2	38.8	39.1	39.0	38.7	38.7	38.7	38.6
	#639	39.7	39.6	39.1	39.0	39.2	38.8	38.4	39.0	39.3	38.9	39.1	39.2	39.2	39.2	38.8	38.8	38.9	39.2	38.8	38.7	38.7	38.9
	#640	39.5	39.2	39.0	39.1	39.1	39.1	38.7	38.7	39.5	39.2	39.7	39.2	39.0	40.0	39.2	38.6	38.7	39.2	38.8	38.6	38.7	38.8

Supplementary Table 6. Rectal temperatures recorded after the challenge.

	Score	Rectal temperature (°C)
Normal	0	<39.5
Mild	1	39.5 – 39.9
Moderate	2	40 – 40.4
Severe	3	40.5 – 40.9
	4	>40.9

Group	Animal number	Clinical signs score																					
		Days post vaccination / Days post challenge																					
		0	2	4	6	9	14	21	28	34/0	36/2	37/3	38/4	39/5	40/6	41/7	42/8	43/9	44/10	45/11	46/12	47/13	48/14
Control	#626	0	1	0	0	0	0	0	0	0	0	1	0	1	0	1	7	3	3	2	1	1	1
	#636	1	0	0	1	0	0	0	0	2	1	2	2	1	2	1	5	6	9	4	7	5	1
	#637	0	0	0	0	0	0	0	0	1	0	1	0	0	1	2	5	3	5	1	2	1	0
	#638	0	0	0	0	0	0	0	0	2	1	2	1	1	0	3	6	7	4	3	2	1	0
	#647	0	0	0	0	0	0	0	0	0	1	1	1	0	1	1	4	4	5	2	0	2	0
Bovi	#627	1	0	1	0	0	0	0	0	0	0	1	0	0	0	2	0	0	1	0	0	0	0
	#628	2	1	2	1	2	0	0	0	1	1	2	2	1	1	0	0	0	1	0	0	0	0
	#629	0	0	0	0	0	0	0	0	1	1	3	1	0	0	0	0	0	0	0	0	0	0
	#632	3	0	0	1	0	0	0	0	1	1	0	0	0	0	0	0	0	0	0	0	0	0
	#635	1	1	0	0	1	0	0	0	0	0	0	0	0	0	0	0	0	0	0	0	0	0
QMV-BVD2*	#630	0	0	0	0	0	0	0	0	0	0	0	1	1	1	0	2	0	0	0	0	0	0
	#633	0	0	0	0	0	0	0	0	1	0	0	0	0	0	0	0	0	0	0	0	0	0
	#639	1	1	0	0	0	0	0	0	0	0	0	0	0	0	0	0	0	0	0	0	0	0
	#640	1	0	0	0	0	0	0	0	1	0	1	0	0	2	0	0	0	0	0	0	0	1

Supplementary Table 7. **Clinical scores recorded after the challenge.**

Supplementary Table 8. **Lung lesion scores**

Treatment	Calf Number	Section	Bronchi	Parenchyma	Septa + Pleura
Control	636	Aa	3	2	2
		Ab	2	1	1
		Ac	0	0	1
Control	647	Ga	3	1	2
		Gb	2	0	2
		Gc	3	1	1
Bovi	635	Ma	1	0	1
		Mb	3	1	2
		Mc	1	0	0
		Md	1	1	1
Control	637	Sa	2	1	2
		Sb	2	0	1
QMV-BVD2*	630	Ya	2	0	0
		Yb	1	0	1
Control	638	AE a	0	0	1
		AE b	2	0	1
		AE c	2	0	1
Bovi	628	AK a	1	0	0
		AK b	1	2	1
		AK c	1	1	1
		AP	3	1	2
QMV-BVD2*	639	AQ a	0	1	0
		AQ b	1	0	2
		AQ c	1	0	1
Bovi	632	AW a	1	1	1
		AW b	3	1	2
Bovi	629	Bl a	2	0	1
		Bl b	2	1	1
Control	626	BO a	0	0	0
		BO b	3	3	3
		BO c	1	0	0
QMV-BVD2*	640	BT a	2	0	2
		BT b	1	0	1
Bovi	627	BZ a	2	0	2
		BZ b	2	1	1
QMV-BVD2*	633	CF a	0	0	1
		CF b	1	1	1
		CF c	1	1	1
